# Respiratory Variability during Exercise: Associations with Motor Severity in Parkinson's Disease

**DOI:** 10.1002/mdc3.70787

**Published:** 2026-08-13

**Authors:** Marcela S. Araújo, Muhammad M. Kathia, Sergiu‐Gabriel Duplea, Julian C. Bommarito, Devin G. McCarthy, Barbara Connolly, Philip J. Millar, Lauro C. Vianna

**Affiliations:** ^1^ NeuroV̇ASQ̇‐Integrative Physiology Laboratory, Faculty of Physical Education University of Brasília Brasília Brazil; ^2^ National Institute of Science and Technology‐INCT, Physical (In)activity and Health CNPq Brasília Brazil; ^3^ Human Cardiovascular Physiology Laboratory, Department of Human Health and Nutritional Sciences, College of Biological Sciences University of Guelph Guelph Ontario Canada; ^4^ Division of Neurology, Department of Medicine McMaster University Hamilton Ontario Canada

**Keywords:** ventilatory control, disease severity, MDS‐UPDRS part III, minute ventilation, cardiopulmonary exercise testing

Respiratory dysfunction is a recognized feature of Parkinson's disease (PD),^1^ encompassing alterations in breathing pattern, reduced respiratory muscle strength, and a blunted hypoxic ventilatory response, even in mild to moderate stages.^2^, ^3^ These abnormalities likely reflect dysfunction across central respiratory networks, chemosensory regulation, and motor activation of respiratory muscles. Because breathing requires precise integration of neural drive and respiratory muscle output, reduced regularity of respiratory muscle activation may contribute to greater breath‐to‐breath variability in ventilation. Thus, progressive motor dysfunction in PD may also be reflected in ventilatory stability. However, the relationship between respiratory variability and motor symptom severity remains unclear. Exercise represents a dynamic physiological challenge to ventilatory control, during which breath‐by‐breath respiratory variability may serve as a marker of underlying dysfunction. Accordingly, we examined whether respiratory variability during maximal exercise is associated with motor symptom severity in PD.

Data were analyzed from 24 participants with idiopathic PD (15 men; 66 ± 8 years; 78 ± 18 kg; 170 ± 10 cm), recruited from the local community for an unrelated study hypothesis.^4^ Exclusion criteria included dementia, stroke, recent smoking, type‐I diabetes, a self‐reported physician diagnosis of autonomic neuropathy, or chronic cardiopulmonary conditions. Participants completed a maximal cardiopulmonary exercise test on an electronically braked cycle ergometer. After a 5‐minute warm‐up, workload increased continuously using individualized ramp protocols until cadence fell below 50 rpm or volitional exhaustion.^4^ All participants were tested during the ON phase of their medications. Respiratory variables were assessed breath‐by‐breath using indirect calorimetry (COSMED Quark, Rome, Italy), including minute ventilation (*V̇*_E_), respiratory frequency (*f*_R_), and tidal volume (*V*_T_). Time‐domain indices of respiratory variability (standard deviation [SD] and root mean square of successive differences [RMSSD]) were calculated across the entire exercise test and reported with and without normalization for the number of respiratory cycles (SD/n and RMSSD/n). Motor symptom severity was assessed using the MDS‐UPDRS Part‐III.

In the full cohort, respiratory variability indices were unrelated to MDS‐UPDRS Part‐III scores (all *p* > 0.52). Patients were subsequently classified according to established motor severity thresholds^5^ into mild (n = 10; MDS‐UPDRS Part III ≤32) and moderate PD (n = 14; MDS‐UPDRS Part III 33–58). Peak VO_2_ was lower in the moderate than in the mild group (*p* = 0.049), whereas peak V̇E, heart rate, and respiratory exchange ratio were similar between groups (all *p* > 0.26). Respiratory variability indices did not differ between the mild and moderate PD groups (all *p* > 0.11). In the mild group, no associations were identified (all *p* > 0.15), and motor symptom scores showed marked clustering, limiting variability for analysis. In patients with moderate motor symptom severity, higher motor symptom scores were positively correlated with RMSSD/n of *V̇*_E_ (*r* = 0.697, *p* = 0.006; Fig. 1A) and *f*_R_ (*r* = 0.698, *p* = 0.006; Fig. 1C). These associations remained after partial correlation analyses controlling for sex, age, and body mass index, with stronger correlations for RMSSD/n of *V̇*_E_ (*r* = 0.84, *p* = 0.001) and *f*_R_ (*r* = 0.77, *p* = 0.005). V̇E RMSSD without normalization for breath count was also associated with motor symptom severity after covariate adjustment (*r* = 0.702, *p* = 0.016), whereas fR RMSSD showed a similar but non‐significant pattern (*r* = 0.54, *p* = 0.088). No associations were observed for VT (*r* = 0.44, *p* = 0.12) or for SD/n‐based indices (*V̇*_E_: *r* = 0.51, *p* = 0.06; *f*_R_: *r* = 0.51, *p* = 0.06; *V*_T_: *r* = 0.27, *p* = 0.34). These findings indicate that greater respiratory variability is associated with motor symptom severity in moderate PD. Whether this instability represents a pathophysiological feature of PD or is preferentially revealed during exercise remains to be established through studies including healthy controls, resting respiratory measurements, and larger cohorts.

**Figure 1:**
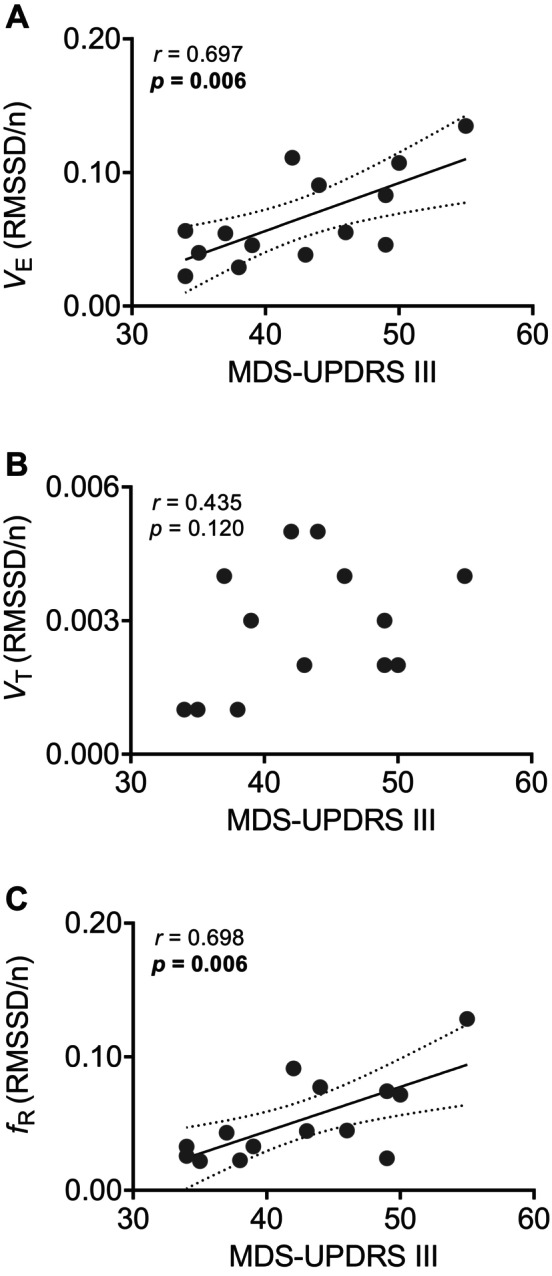
Associations between respiratory variability during exercise and motor symptom severity in patients with Parkinson's disease with moderate motor impairment (n = 14). Scatter plots show the relationships between MDS‐UPDRS Part III scores and RMSSD normalized by the number of breaths (RMSSD/n) for minute ventilation (*V̇*_E_; Top panel), tidal volume (*V*_T_; middle panel), and respiratory frequency (*f*_R_; Bottom panel) during a maximal progressive cardiopulmonary exercise test.

In summary, in patients with moderate PD, greater motor symptom severity is associated with increased short‐term, breath‐by‐breath respiratory variability during exercise, as captured by RMSSD. These findings suggest that respiratory variability during progressive maximal exercise may represent a potentially informative marker of disease‐related dysfunction in PD.

## Author Roles

(1) Research Project: A. Design, B. Execution, C. Data Collection, D. Supervision; (2) Statistical Analysis: A. Execution, B. Interpretation, C. Review and Critique; (3) Manuscript Preparation: A. Writing of the First Draft, B. Review and Critique.

M.S.A.: 1A, 1B, 2A, 2B, 2C, 3A, 3B.

M.M.K.: 1C, 2B, 3B.

S.G.D.: 1C, 2B, 3B.

J.C.B.: 1C, 2B, 3B.

D.G.M.: 1C, 2B, 3B.

B.C.: 1C, 2B, 3B.

P.J.M.: 1A, 1C, 1D, 2B, 2C, 3B.

L.C.V: 1A, 1B, 1D, 2A, 2B, 2C, 3A, 3B.

## Disclosures

**Ethical Compliance Statement:** The study was approved by the University of Guelph Research Ethics Board, and all participants provided written informed consent. The study was registered on ClinicalTrials.gov (NCT03940261). We confirm that we have read the Journal's position on issues involved in ethical publication and affirm that this work is consistent with those guidelines.

## Financial Disclosures and Conflicts of Interest

Author disclosures are available in the Supporting Information.

## Supporting information


**Data S1.** Coi_disclosure_all.

## Data Availability

The data that support the findings of this study are available from the corresponding author upon reasonable request.
